# Association of Ischemic Stroke, Major Bleeding, and Other Adverse Events With Warfarin Use vs Non–vitamin K Antagonist Oral Anticoagulant Use in Patients With Atrial Fibrillation With a History of Intracranial Hemorrhage

**DOI:** 10.1001/jamanetworkopen.2020.6424

**Published:** 2020-06-01

**Authors:** Chuan-Tsai Tsai, Jo-Nan Liao, Chern-En Chiang, Yenn-Jiang Lin, Shih-Lin Chang, Li-Wei Lo, Yu-Feng Hu, Ta-Chuan Tuan, Fa-Po Chung, Tze-Fan Chao, Gregory Y. H. Lip, Shih-Ann Chen

**Affiliations:** 1 Division of Cardiology, Department of Medicine, Taipei Veterans General Hospital, Taipei, Taiwan; 2 Institute of Clinical Medicine and Cardiovascular Research Center, National Yang-Ming University, Taipei, Taiwan; 3 General Clinical Research Center, Taipei Veterans General Hospital, Taipei, Taiwan; 4 Liverpool Centre for Cardiovascular Science, University of Liverpool and Liverpool Heart and Chest Hospital, Liverpool, United Kingdom; 5 Aalborg Thrombosis Research Unit, Department of Clinical Medicine, Aalborg University, Aalborg, Denmark

## Abstract

**Importance:**

Current guidelines recommend the use of non–vitamin K antagonist oral anticoagulants (NOACs) for stroke prevention in patients with atrial fibrillation (AF). Data regarding warfarin sodium use compared with NOAC use in patients with AF with a history of intracranial hemorrhage (ICH) are limited.

**Objective:**

To compare the clinical outcomes of warfarin use and NOAC use in patients with AF with a history of ICH using a nationwide cohort with AF.

**Design, Setting, and Participants:**

A nationwide cohort study from January 1, 2012, to December 31, 2016, was performed using data from the Taiwan National Health Insurance Research Database. The dates of analysis were July 1 to September 1, 2019. The study population comprised patients with AF with a history of ICH and a CHA_2_DS_2_-VASc score (congestive heart failure, hypertension, age ≥75 years [doubled], diabetes, prior stroke/transient ischemic attack/thromboembolism [doubled], vascular disease [prior myocardial infarction, peripheral artery disease], age 65-74 years, sex category [female]) of at least 1 for men or at least 2 for women who had received warfarin or NOACs. The clinical outcomes were examined using Cox proportional hazards regression analyses among the study population before and after propensity score matching.

**Exposures:**

Oral anticoagulation with warfarin or NOACs.

**Main Outcomes and Measures:**

The clinical outcomes measured were all-cause mortality, ischemic stroke, ICH, major bleeding, and adverse events.

**Results:**

The study cohort included 4540 patients (mean [SD] age, 76.0 [10.5] years; 2653 men [58.4%]), with 1047 patients receiving warfarin (mean [SD] age, 75.1 [11.4] years; 571 men [54.5%]) and 3493 patients receiving NOACs (mean [SD] age, 76.3 [10.2] years; 2082 men [59.6%]). Compared with warfarin use, NOAC use was associated with statistically significantly lower risk of all-cause mortality (adjusted hazard ratio [aHR], 0.517; 95% CI, 0.457-0.585), ICH (aHR, 0.556; 95% CI, 0.389-0.796), and major bleeding (aHR, 0.645; 95% CI, 0.525-0.793), whereas the rate of ischemic stroke was similar in the 2 groups (aHR, 0.879; 95% CI, 0.678-1.141). These results were generally consistent after propensity score matching among 973 patients in each group.

**Conclusions and Relevance:**

Among patients with AF with prior ICH, NOAC use was associated with lower rates of ICH and major bleeding compared with warfarin use, whereas the rate of ischemic stroke was similar in the 2 groups. Among patients with AF with prior ICH, NOACs could be the preferred choice for stroke prevention.

## Key Points

**Question:**

Are non–vitamin K antagonist oral anticoagulants (NOACs) associated with greater clinical benefit than warfarin sodium among patients with atrial fibrillation with a history of intracranial hemorrhage?

**Findings:**

In this cohort study, 4540 patients were identified with prior intracranial hemorrhage who received warfarin or NOACs. The use of NOACs was associated with statistically significantly lower rates of all-cause mortality, intracranial hemorrhage, and major bleeding compared with the use of warfarin, whereas the rate of ischemic stroke was similar in the 2 groups.

**Meaning:**

Among patients with atrial fibrillation with prior intracranial hemorrhage, NOACs could be the preferred choice for stroke prevention.

## Teaser

This cohort study in Taiwan compares the clinical outcomes of warfarin use and non–vitamin K antagonist oral anticoagulant use in patients with atrial fibrillation with a history of intracranial hemorrhage using a nationwide cohort with atrial fibrillation.

## Introduction

Atrial fibrillation (AF) is the most common sustained arrhythmia and is an important cause of ischemic stroke.^1^ In large randomized studies and meta-analysis,^2,3,4,5,6,7^ non–vitamin K antagonist oral anticoagulants (NOACs) were noninferior to warfarin sodium for comparable risk reduction of ischemic stroke and less bleeding. Therefore, current guidelines recommend NOAC use in patients with AF.^8,9,10^

Intracranial hemorrhage (ICH) is a serious complication of oral anticoagulant use, and overall mortality is high once ICH occurs.^11^ The risk of ischemic stroke is even higher in patients with AF who survive after ICH compared with those without ICH.^12^ In observational cohorts, warfarin use was associated with lower risk of ischemic stroke in patients with AF with prior ICH,^13^ but there are no solid trial data on NOAC use in this patient population because patients with prior ICH were excluded from the relevant randomized trials.^3,4,5,6^ Therefore, it is unknown if the use of NOACs is associated with lower rates of ischemic stroke and other adverse events in this population. In the present study, we used a nationwide cohort in Taiwan to compare the clinical outcomes of warfarin use and NOAC use in patients with AF with a history of ICH.

## Methods

This nationwide cohort study used the Taiwan National Health Insurance Research Database (NHIRD) provided by the Health and Welfare Data Science Center, Ministry of Health and Welfare, Taiwan. The National Health Insurance system is a mandatory universal health insurance program that offers comprehensive medical care coverage to all residents of Taiwan. The NHIRD consists of detailed health care data from more than 23 million enrollees, representing more than 99% of Taiwan’s population. In this cohort data set, patients’ original identification numbers were encrypted to protect their privacy, but the encryption was consistent so that a patient’s claims linkage was feasible within the National Health Insurance database and could be followed continuously. Details about the NHIRD have been described in previous studies.^14,15,16^ The present study was approved by the institutional review board at Taipei Veterans General Hospital, Taipei, Taiwan. Informed consent was waived because anonymous data were used. This study followed the Strengthening the Reporting of Observational Studies in Epidemiology (STROBE) reporting guideline.

### Study Population

From January 1, 2012, to December 31, 2016, a total of 162 124 patients 20 years or older newly diagnosed as having AF were identified from the NHIRD. The analysis was conducted from July 1 to September 1, 2019. The diagnosis of AF was identified using the *International Classification of Diseases*, *Ninth Revision*, *Clinical Modification* (*ICD-9-CM*) code 427.31, which was registered by physicians for their patients. The diagnostic accuracy of AF using this definition in the NHIRD has been validated previously.^17^ The study population comprised 4540 patients with AF with a history of ICH and a CHA_2_DS_2_-VASc score (congestive heart failure, hypertension, age ≥75 years [doubled], diabetes, prior stroke/transient ischemic attack/thromboembolism [doubled], vascular disease [prior myocardial infarction or peripheral artery disease], age 65-74 years, sex category [female]) of at least 1 for men or at least 2 for women who had received warfarin (n = 1047) or NOACs (n = 3493 [1430 received dabigatran, 1686 received rivaroxaban, and 377 received apixaban]). The CHA_2_DS_2_-VASc score is based on 10 possible points, with higher scores indicating higher risk. The mean (SD) interval between the diagnosis of AF and a history of ICH was 5.9 (5.4) years. At the time of prior ICH, among 4540 patients, 4 (0.09%) were receiving NOACs, 494 (10.9%) were receiving warfarin, 1438 (31.7%) were receiving antiplatelet drugs, and 2697 (59.4%) were receiving no antithrombotic therapy. A flowchart of patient enrollment and the study design is shown in the eFigure in the Supplement.

### CHA_2_DS_2_-VASc Score, HAS-BLED Score, and Clinical End Points

The CHA_2_DS_2_-VASc score was calculated for each patient by assigning 1 point each for congestive heart failure, hypertension, diabetes, vascular disease (prior myocardial infarction or peripheral artery disease), age between 65 and 74 years, and female sex and 2 points each for prior stroke/transient ischemic attack (TIA) and age 75 years or older.^18^ The CHA_2_DS_2_-VASc score is recommended by American and European guidelines to estimate the risk of ischemic stroke in patients with AF and to guide antithrombotic therapies for stroke prevention.^9,10,19^ The HAS-BLED (hypertension, abnormal kidney or liver function, stroke, bleeding history, age 65 years or older, and antiplatelet drug or alcohol use) score assesses bleeding risk in patients with AF.^20^ In this study, the HAS-BLED score ranged from 0 to 8, with higher scores indicating greater risk of bleeding. It was calculated by assigning 1 point each for hypertension, abnormal kidney or liver function, stroke, bleeding history, age 65 years or older, and antiplatelet drug or alcohol use.^20^ Because the international normalized ratio (INR) for warfarin was not available in the NHIRD, labile INR was excluded from scoring in the present study, consistent with prior registry studies.^21,22^ Abnormal kidney or liver function was defined by *ICD-9-CM* codes rather than by laboratory data. Herein, the CHA_2_DS_2_-VASc and HAS-BLED scores were used to represent the risk of ischemic stroke and major bleeding, respectively, in the study population, and the CHA_2_DS_2_-VASc score was included as a covariate in the Cox proportional hazards regression analyses.

Clinical end points were all-cause mortality, ischemic stroke, ICH, major bleeding, and adverse events. Ischemic stroke was identified using *ICD-9-CM* codes with concomitant imaging studies, including computed tomography or magnetic resonance imaging. The accuracy of ischemic stroke diagnosis in the NHIRD is approximately 94%.^23^ Another validation study^24^ demonstrated that the diagnostic accuracy of ischemic stroke in the NHIRD is high, with a positive predictive value of 88.4% and a sensitivity of 97.3%. Major bleeding was defined as ICH or bleeding from the gastrointestinal, genitourinary, or respiratory tract requiring hospitalization.

### Statistical Analysis

Continuous variables in each group (warfarin vs NOACs) were expressed as means (SDs), and categorical variables were expressed as proportions. Differences between continuous values were assessed using an unpaired 2-tailed *t* test, and differences between nominal variables were compared using the χ^2^ test. The incidence rates of events were calculated by dividing the number of events across the entire study period by person-years at risk.

#### Primary Analysis Among the Study Cohort Without Propensity Score Matching

The rates of clinical events with warfarin use vs NOAC use were compared among the unmatched cohort using multivariable Cox proportional hazards regression analysis adjusted for variables that were statistically significantly different between the 2 groups, including age, sex, hyperlipidemia, abnormal kidney function, anemia, use of antiplatelet drugs, and CHA_2_DS_2_-VASc score. Subgroup analysis according to age (65-74 vs ≥75 years), sex, prior stroke/TIA, heart failure, abnormal kidney function, and abnormal liver function was also performed with patients receiving warfarin vs those receiving NOACs in the unmatched cohort.

#### Propensity Score Matching Analysis (Sensitivity Analysis)

Propensity scores were calculated for the likelihood of receiving warfarin vs NOACs by multivariable logistic regression analyses conditional on all baseline covariates listed in the Table. Patients in the warfarin group were then matched 1:1 to patients in the NOAC group on the basis of the closest propensity score for the use of NOACs within a threshold of ±0.01 using a greedy algorithm. If more than 1 patient in the NOAC group could be matched to the corresponding patient in the warfarin group, 1 patient from the NOAC group was randomly selected without repeat sampling. After propensity score matching, univariate Cox proportional hazards regression analysis was performed to compare the rates of clinical events with warfarin vs NOACs. Cumulative incidence curves of events were plotted using the Kaplan-Meier method among the propensity score–matched cohort with statistical significance assessed by the log-rank test. Statistical significance was set at 2-tailed *P* < .05.

**Table.** Baseline Characteristics of the Whole Population and the Matched Population

Variable	Whole population				Matched population		
	All (N = 4540)	Warfarin (n = 1047)	NOACs (n = 3493)	*P* value	Warfarin (n = 973)	NOACs (n = 973)	*P* value
Age, mean (SD), y	76.0 (10.5)	75.1 (11.4)	76.3 (10.2)	.002	75.5 (11.1)	75.7 (10.7)	.65
Age, No. (%), y							
65-74	1154 (25.4)	250 (23.9)	904 (25.9)	.19	233 (23.9)	246 (25.3)	.49
≥75	2743 (60.4)	595 (56.8)	2148 (61.5)	.007	566 (58.2)	587 (60.3)	.33
Male, No. (%)	2653 (58.4)	571 (54.5)	2082 (59.6)	.004	543 (55.8)	502 (51.6)	.06
Comorbidities, No. (%)							
Hypertension	4272 (94.1)	981 (93.7)	3291 (94.2)	.53	911 (93.6)	911 (93.6)	>.99
Diabetes	2277 (50.2)	522 (49.9)	1755 (50.2)	.83	478 (49.1)	509 (52.3)	.16
Prior stroke/TIA	3393 (74.7)	705 (67.3)	2688 (77.0)	<.001	673 (69.2)	678 (69.7)	.81
Heart failure	2495 (55.0)	631 (60.3)	1864 (53.4)	<.001	573 (58.9)	584 (60.0)	.61
Vascular disease	843 (18.6)	220 (21.0)	623 (17.8)	.03	192 (19.7)	191 (19.6)	.96
Myocardial infarction	472 (10.4)	140 (13.4)	332 (9.5)	<.001	105 (10.8)	107 (11.0)	.90
Peripheral artery disease	422 (9.3)	97 (9.3)	325 (9.3)	.97	93 (9.6)	92 (9.5)	.94
COPD	2399 (52.8)	544 (52.0)	1855 (53.1)	.51	514 (52.8)	534 (54.9)	.36
Hyperlipidemia	2633 (58.0)	556 (53.1)	2077 (59.5)	<.001	520 (53.4)	550 (56.5)	.17
Autoimmune diseases	446 (9.8)	100 (9.6)	346 (9.9)	.74	92 (9.5)	92 (9.5)	>.99
Cancer	744 (16.4)	161 (15.4)	583 (16.7)	.31	151 (15.5)	165 (17.0)	.39
Abnormal kidney function	1135 (25.0)	338 (32.3)	797 (22.8)	<.001	280 (28.8)	294 (30.2)	.487
Abnormal liver function	1581 (34.8)	342 (32.7)	1239 (35.5)	.091	327 (33.6)	350 (36.0)	.27
Anemia	947 (20.9)	301 (28.7)	646 (18.5)	<.001	248 (25.5)	246 (25.3)	.92
History of bleeding	4540 (100)	1047 (100)	3493 (100)	NA	973 (100)	973 (100)	NA
Alcohol excess/abuse	177 (3.9)	41 (3.9)	136 (3.9)	.97	39 (4.0)	35 (3.6)	.64
Use of antiplatelet drugs, No. (%)	510 (11.2)	197 (18.8)	313 (9.0)	<.001	150 (15.4)	150 (15.4)	>.99
Use of NSAIDs, No. (%)	156 (3.4)	40 (3.8)	116 (3.3)	.44	38 (3.9)	40 (4.1)	.82
CHA_2_DS_2_-VASc score, mean (SD)	5.55 (1.67)	5.43 (1.81)	5.59 (1.63)	.009	5.44 (1.79)	4.59 (1.71)	.06
HAS-BLED score, mean (SD)	4.31 (1.05)	4.31 (1.15)	4.30 (1.02)	.81	4.29 (1.15)	4.35 (1.13)	.25
Propensity score, mean (SD)	NA	NA	NA	NA	0.74 (0.10)	0.74 (0.10)	.86

Abbreviations: CHA_2_DS_2_-VASc, congestive heart failure, hypertension, age at least 75 years (doubled), diabetes, prior stroke/TIA/thromboembolism (doubled), vascular disease (prior myocardial infarction or peripheral artery disease), age 65 to 74 years, sex category (female); COPD, chronic obstructive pulmonary disease; HAS-BLED, hypertension, abnormal kidney or liver function, stroke, bleeding history, age 65 years or older, and antiplatelet drug or alcohol use; NA, not applicable; NOACs, non–vitamin K antagonist oral anticoagulants; NSAID, nonsteroidal anti-inflammatory drug; TIA, transient ischemic attack.

## Results

The study cohort included 4540 patients (mean [SD] age, 76.0 [10.5] years; 2653 men [58.4%]), with 1047 patients receiving warfarin (mean [SD] age, 75.1 [11.4] years; 571 men [54.5%]) and 3493 patients receiving NOACs (mean [SD] age, 76.3 [10.2] years; 2082 men [59.6%]). The mean (SD) CHA_2_DS_2_-VASc score was 5.55 (1.67), with hypertension (4272 [94.1%]) being the most common comorbidity (Table). Before propensity score matching, patients receiving NOACs were older than patients receiving warfarin and had higher prevalence of men, prior stroke/TIA, and hyperlipidemia. Compared with patients receiving NOACs, patients receiving warfarin had higher prevalence of heart failure, vascular disease, abnormal kidney function, anemia, and use of antiplatelet drugs. The CHA_2_DS_2_-VASc score was higher in patients receiving NOACs vs warfarin (mean [SD], 5.59 [1.63] vs 5.43 [1.81]; *P* = .009), whereas there was no statistically significant difference in HAS-BLED scores in patients receiving NOACs vs warfarin (mean [SD], 4.30 [1.02] vs 4.31 [1.15]; *P* = .81). After propensity score matching, 973 matched patients whose baseline characteristics did not differ significantly remained in each group (Table).

### Risk of Clinical End Points in the Original Cohort Before Propensity Score Matching

**Figure 1.**
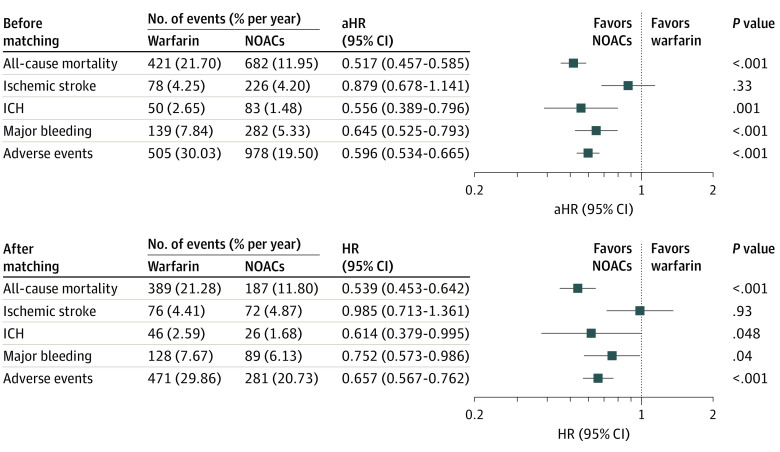
Clinical End Points of Non–vitamin K Antagonist Oral Anticoagulant (NOAC) Use Compared With Warfarin Use Before and After Propensity Score Matching Before matching, multivariable Cox proportional hazards regression analysis showed that NOAC use was associated with lower rates of all-cause mortality, ICH, major bleeding, and adverse events and a similar rate of ischemic stroke compared with warfarin sodium use. After matching, the principal findings were generally consistent with those before matching. aHR indicates adjusted hazard ratio; HR, hazard ratio; and ICH, intracranial hemorrhage.

The annual risk of clinical end points and comparison between warfarin and NOACs are shown in Figure 1. All-cause mortality occurred in 421 patients receiving warfarin and in 682 patients receiving NOACs, with an annual incidence of 21.70% (95% CI, 19.63%-23.77%) and 11.95% (95% CI, 11.05%-12.85%), respectively. In addition, 78 patients receiving warfarin and 226 patients receiving NOACs had ischemic stroke, with an annual incidence of 4.25% (95% CI, 3.31%-5.19%) and 4.20% (95% CI, 3.65%-4.75%), respectively. Multivariable Cox proportional hazards regression analysis showed that NOAC use was associated with lower risk of all-cause mortality (adjusted hazard ratio [aHR], 0.517; 95% CI, 0.457-0.585; *P* < .001) and similar risk of ischemic stroke (aHR, 0.879; 95% CI, 0.678-1.141; *P* = .33) compared with warfarin use.

Regarding safety end points, 50 patients receiving warfarin and 83 patients receiving NOACs had ICH, with an annual incidence of 2.65% (95% CI, 1.92%-3.38%) and 1.48% (95% CI, 1.16%-1.80%), respectively (Figure 1). Moreover, 139 patients receiving warfarin and 282 patients receiving NOACs experienced major bleeding, with an annual incidence of 7.84% (95% CI, 6.54%-9.14%) and 5.33% (95% CI, 4.71%-5.95%), respectively. Multivariable Cox proportional hazards regression analysis showed that NOAC use was associated with lower risk of ICH (aHR, 0.556; 95% CI, 0.389-0.796; *P* = .001) and major bleeding (aHR, 0.645; 95% CI, 0.525-0.793; *P* < .001) compared with warfarin use. Overall, NOAC use was associated with decreased risk of adverse events (aHR, 0.596; 95% CI, 0.534-0.665; *P* < .001) compared with warfarin use.

### Risk of Clinical End Points in the Cohort After Propensity Score Matching

**Figure 2.**
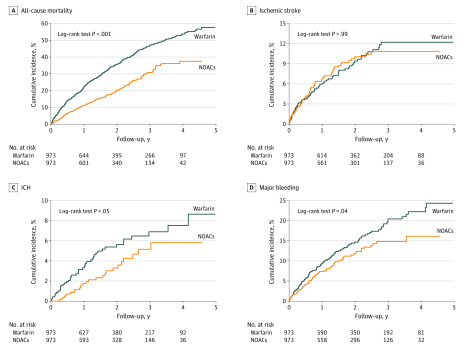
Cumulative Incidence Curves of Clinical End Points Among the Propensity Score–Matched Cohort Using the Kaplan-Meier Method Cumulative incidence curves for all-cause mortality (A), ICH (C), and major bleeding (D) were lower for non–vitamin K antagonist oral anticoagulant (NOAC) use compared with warfarin sodium use. Cumulative incidence curves for ischemic stroke were similar for warfarin use and NOAC use (B). ICH indicates intracranial hemorrhage.

The annual risk of clinical end points and comparison between warfarin and NOACs after propensity score matching are shown in Figure 1. The principal findings were generally consistent with those of multivariable Cox proportional hazards regression analysis performed among the cohort before propensity score matching. Compared with warfarin use, NOAC use was associated with statistically significantly lower risk of all-cause mortality (HR, 0.539; 95% CI, 0.453-0.642; *P* < .001), ICH (HR, 0.614; 95% CI, 0.379-0.995; *P* = .048), and major bleeding (HR, 0.752; 95% CI, 0.573-0.986; *P* = .04), whereas the risk of ischemic stroke was similar (HR, 0.985; 95% CI, 0.713-1.361; *P* = .93) (Figure 1). Cumulative incidence curves of clinical end points among the propensity score–matched cohort are shown in Figure 2.

### Subgroup Analysis

**Figure 3.**
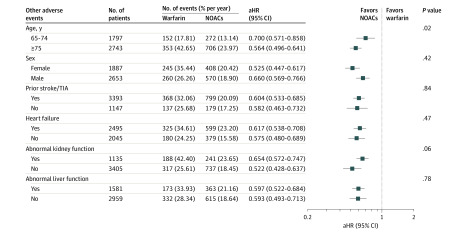
Risk of Adverse Events Associated With Non–vitamin K Antagonist Oral Anticoagulant (NOAC) Use Compared With Warfarin Use in Different Subgroups Subgroup analysis according to age, sex, prior stroke/TIA, heart failure, abnormal kidney function, and abnormal liver function was performed. Compared with warfarin sodium use, NOAC use was associated with a lower rate of adverse events in all subgroups, which was more evident among patients 75 years or older than those aged 65 to 74 years. aHR indicates adjusted hazard ratio; and TIA, transient ischemic attack.

Figure 3 compares adverse events in different subgroups for warfarin vs NOACs. The use of NOACs was consistently associated with a lower rate of adverse events (interaction *P* > .05 in all subgroups except for age), which was more evident among patients 75 years or older (aHR, 0.564; 95% CI, 0.496-0.641) than those younger than 75 years (aHR, 0.700; 95% CI, 0.571-0.858) (interaction *P* = .02).

## Discussion

This nationwide cohort study compared the rates of all-cause mortality, ischemic stroke, ICH, major bleeding, and adverse events associated with warfarin use vs NOAC use in patients with AF with a history of ICH, with 2 main findings. First, compared with warfarin use, NOAC use was associated with statistically significantly lower risk of all-cause mortality, ICH, and major bleeding, whereas the rate of ischemic stroke was similar in the 2 groups. Second, in subgroup analysis, NOAC use was consistently associated with a lower rate of adverse events than warfarin use.

The use of oral anticoagulants for stroke prevention in patients with AF should balance the risk of stroke if left untreated and increased bleeding risk if anticoagulated.^25^ Bleeding risk is a main reason why physicians withhold oral anticoagulant therapy in patients with AF. In the Outcomes Registry for Better Informed Treatment of Atrial Fibrillation (ORBIT-AF), which studied 10 130 patients with AF, 13.1% had contraindications to oral anticoagulation documented by a health care professional, with a prior bleeding event reported in 27.7% of these patients, high bleeding risk in 18.0%, and prior ICH in 5.0%.^26^ Improved safety of NOACs compared with warfarin, especially a 52% lower risk of ICH,^2^ has changed the landscape of stroke prevention in AF. Data from 2 global registry studies demonstrated that overall oral anticoagulant use statistically significantly increased from 57.4% in 2010-2011 to 71.1% in 2014-2015 in the Global Anticoagulant Registry in the FIELD–Atrial Fibrillation (GARFIELD-AF)^27^ and from 52.4% in 2008 to 60.7% in 2014 in the National Cardiovascular Data Registry PINNACLE registry.^28^ These statistically significant increased rates of oral anticoagulant use were largely attributable to greater prescription of NOACs (from 4.2% to 37% during the study period in GARFIELD-AF and from 0% to 25.8% during the study period in the National Cardiovascular Data Registry PINNACLE registry), although regional differences are evident.^27,28,29^ In Asia, the rates of appropriate prescription of oral anticoagulants have also statistically significantly increased, with NOACs commonly prescribed since they became available.^30,31^ In Taiwan, oral anticoagulant prescription rates increased from 13.6% in 2008 to 35.6% in 2015, and NOACs accounted for 73% of overall oral anticoagulants prescribed for patients with incident AF in a 2018 study.^31^

Despite increasing prescription of NOACs worldwide, some high-risk populations were excluded from the pivotal trials^3,4,5,6^ of warfarin vs NOACs (eg, patients with AF with prior ICH). In our study, we compared clinical event rates associated with warfarin use and NOAC use in this high-risk population with a mean CHA_2_DS_2_-VASc score of 5.55 and a mean HAS-BLED score of 4.31. In a previous report, the annual ICH risk in Taiwan was 1.41% for patients with AF treated with warfarin and 0.70% to 0.74% for patients with AF treated with NOACs.^32^ In the present study, the annual risk of recurrent ICH for patients with AF with a history of ICH was 2.65% for warfarin and 1.48% for NOACs. These observations show that patients with AF with prior ICH are at high risk for recurrent ICH despite the type of oral anticoagulant received. However, our results demonstrate that NOAC use is still associated with a statistically significantly lower rate of ICH (aHR, 0.56) and major bleeding (aHR, 0.65) compared with warfarin use and thus should be a more favorable choice for stroke prevention in this population. The 44% lower rate of ICH we observed for NOAC use compared with warfarin use is close to that observed in the pooled analysis of the 4 NOAC trials^3,4,5,6^ (52%) that only enrolled patients with AF without a history of ICH. Therefore, the present study provides important data that were lacking in the randomized trials^3,4,5,6^ and should be complementary to the current literature on stroke prevention using NOACs.

In addition to the lower rates of ICH and major bleeding associated with NOAC use, we observed a lower rate of all-cause mortality with NOACs compared with warfarin, which was not demonstrated in 3 of the NOAC trials,^4,5,6^ with the exception being apixaban use in the Apixaban for Reduction in Stroke and Other Thromboembolic Events in Atrial Fibrillation (ARISTOTLE) study.^2^ In contrast to the randomized trials, observational studies^32,33,34^ showed a statistically significantly lower mortality rate with NOAC use compared with warfarin use. However, a possible explanation for this difference is that some deaths in observational cohorts may be from unrecorded fatal strokes or ICH because not all outcomes are adjudicated and postmortem examinations are not mandated. Therefore, lower mortality with NOAC use may in part be associated with lower risk of stroke and ICH. However, some unmeasured confounders associated with warfarin or NOAC prescription that were also associated with mortality were likely present and may have confounded the analyses.

### Limitations

Our study has some limitations. First, this nationwide cohort study was based on the Taiwan NHIRD and may be limited by coding errors. However, diagnoses of AF, stroke, and other comorbidities in this data set are well validated.^17,23,24,35^ Second, detailed information on prior ICH events was not available, such as location on imaging, severity, and functional disabilities, all of which might influence the choice of oral anticoagulants. Third, data were lacking on the INR and on time in the therapeutic range of warfarin. Fourth, although we tried to adjust for baseline differences between the warfarin and NOAC groups using multivariable Cox proportional hazards regression analyses and propensity score matching, some selection bias might have remained that could have altered the outcomes.

## Conclusions

Among patients with AF with a history of ICH, NOAC use was associated with lower rates of ICH and major bleeding compared with warfarin use, whereas the rate of ischemic stroke was similar in the 2 groups. In this high-risk population with AF, NOACs could be the preferred choice for stroke prevention.

## Notes

Supplement. **eFigure.** Flowchart of the Enrollment of Study Patients
