# Supplementary material for: Association of Ischemic Stroke, Major Bleeding, and Other Adverse Events With Warfarin Use vs Non–vitamin K Antagonist Oral Anticoagulant Use in Patients With Atrial Fibrillation With a History of Intracranial Hemorrhage
Source: JAMA Netw Open. 2020 Jun 1;3(6):e206424. doi: 10.1001/jamanetworkopen.2020.6424 (PMC7265096; doi:10.1001/jamanetworkopen.2020.6424)
Supplement: Supplement. — eFigure. Flowchart of the Enrollment of Study Patients [file jamanetwopen-3-e206424-s001.pdf]

Supplementary Online Content

Tsai C-T, Liao J-N, Chiang C-E, et al. Association of ischemic stroke, major bleeding, and other adverse events with warfarin use vs non–vitamin K antagonist oral anticoagulant use in patients with atrial fibrillation with a history of intracranial hemorrhage. *JAMA Netw Open*. 2020;3(6):e206424. doi:10.1001/jamanetworkopen.2020.6424

eFigure. Flowchart of the Enrollment of Study Patients

This supplementary material has been provided by the authors to give readers additional information about their work.

© 2020 Tsai C-T et al. *JAMA Network Open*.

eFigure. Flowchart of the Enrollment of Study Patients

From January 1, 2012 to December 31, 2016, 162,124 patients aged ≥20 years newly diagnosed with AF were identified from NHIRD. The study population comprised 4,540 patients with a history of ICH and a CHA_2DS_2-VASc score ≥ 1 for males or ≥ 2 for females who had received warfarin (n = 1,047) or NOACs (n = 3,493; 1,430 with dabigatran, 1,686 with rivaroxaban and 377 with apixaban). To balance the baseline differences between patients treated with warfarin or NOACs, the propensity match analysis was performed, and 973 patients remained in each group.

AF = atrial fibrillation; ICH = intracranial hemorrhage; NHIRD = National Health Insurance Research Database; NOACs = non-vitamin K antagonist oral anticoagulants

© 2020 Tsai C-T et al. *JAMA Network Open*.

eFigure. Flowchart of the Enrollment of Study Patients

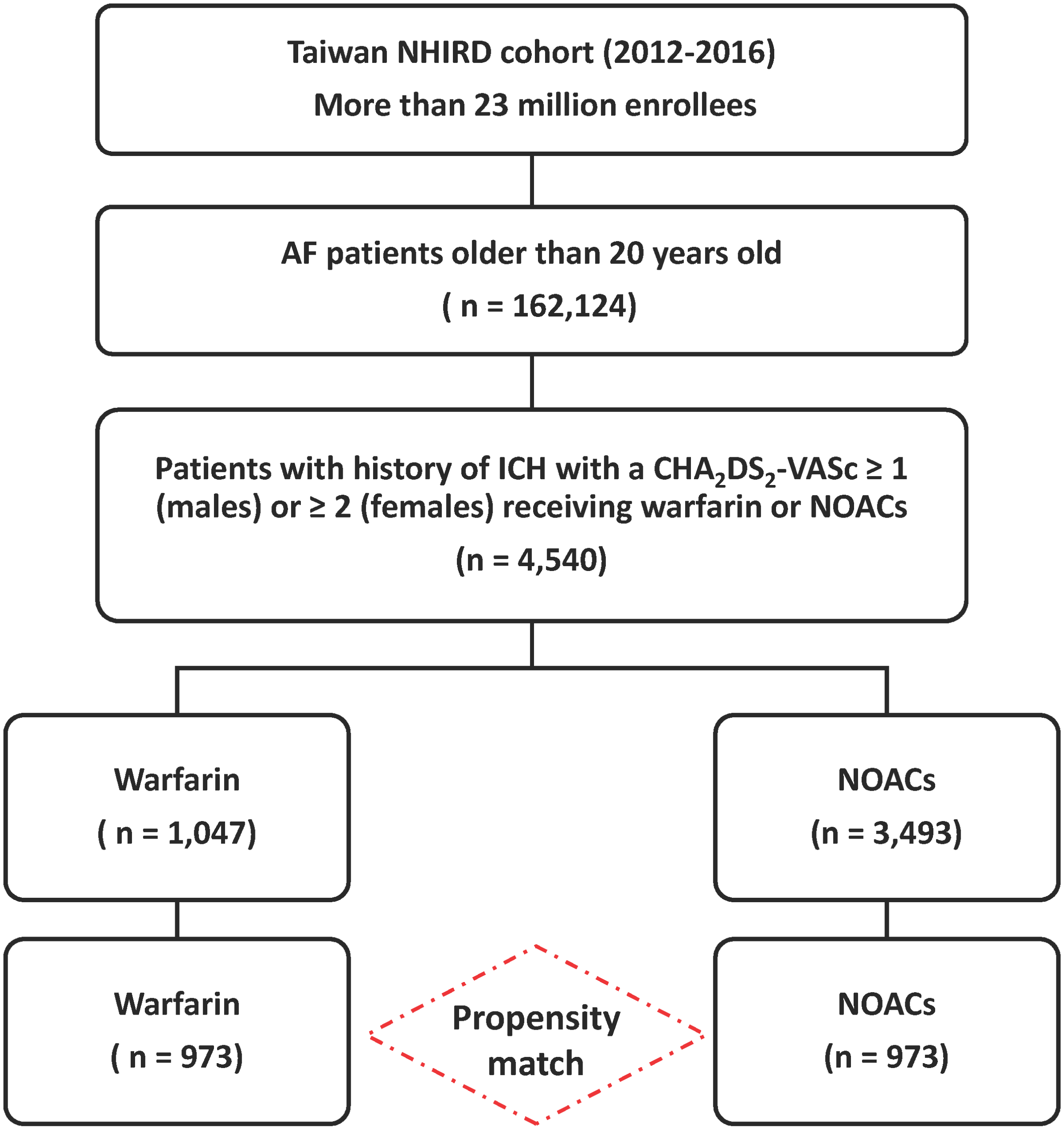

© 2020 Tsai C-T et al. *JAMA Network Open*.
